# Woven coronary artery with acquired etiology: the natural history documented by serial angiography and optical coherence tomography

**DOI:** 10.1186/s12872-024-04025-4

**Published:** 2024-07-12

**Authors:** Jiannan Li, Xiaoli Wang, Chen Liu, Peng Zhou, Hanjun Zhao

**Affiliations:** Department of Cardiology, Fuwai Hospital, National Center for Cardiovascular Diseases, Peking Union Medical College and Chinese Academy of Medical Sciences, Beijing, China

**Keywords:** Woven coronary artery, Optical coherence tomography, Coronary angiography

## Abstract

Woven coronary artery (WCA) is a rare anomaly and its etiology remains speculative. Both congenital and acquired factors are considered to be concerned with the pathogenesis. In a 35-year-old man, the tissue characteristics of WCA were evaluated by optical coherence tomography. Serial coronary angiography indicated that acquired factor is the cause, and thrombus recanalization is the most likely pathological mechanism.

**Supplementary Information:**

The online version contains supplementary material available at 10.1186/s12872-024-04025-4.

## Introduction

Woven coronary artery (WCA) is a rare anomaly and the exact etiology is still unknown [1]. It is angiographically characterized by that the epicardial coronary artery is divided into multiple thin channels at the proximal segment and converged together in the distal segment, with a TIMI III distal blood flow [2]. Since the first case report, fewer than 40 cases of WCA have been reported [1, 3]. Both congenital and acquired origins were considered for WCA. However, the etiology is speculative as lack of longitudinal natural history data. Herein, we report through serial coronary angiography (CAG) and final optical coherence tomography (OCT) a case with WCA in the left descending artery (LAD) in a young man.

## Case presentation

**Fig. 1:**
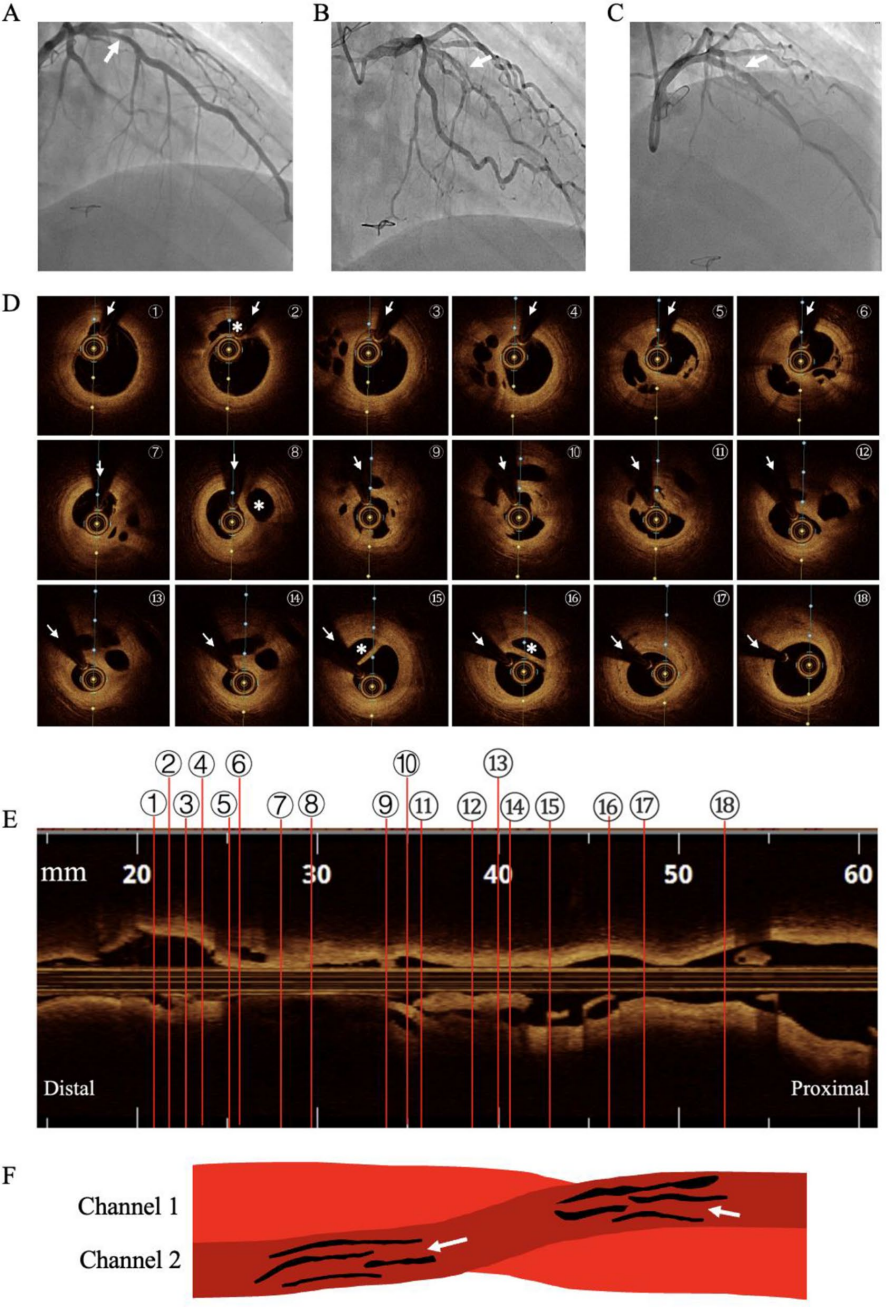
Coronary angiogram and intravascular imaging. (**A**) Coronary angiogram 7 years ago. Arrow shows mild stenosis in the proximal segment of LAD. (**B**) Coronary angiogram during re-hospitalization. Arrow shows twisting channels in the proximal and middle segment of LAD. (**C**) Coronary angiogram after stent implantation in the proximal and middle segment of LAD (arrow). (**D**) OCT images from the distal to proximal segments of LAD. Arrows show shadows of the guidewire. There were two main channels and the circle in the larger lumen represents OCT catheter. Asterisks show a smaller lumen originated and converged after emitting multi-microchannels. Communications were not found between the larger and the smaller channels (diameters about 2000 μm and 1000 μm, respectively), but exist among multi-microchannels which originated from the smaller lumen. (**E**) Longitudinal view corresponding to D. (**F**) Diagrammatic view of the OCT finding in D and F. Channels 1 and 2 are separated with no communication. Arrows show microchannels in Channel 2 and communications exist among the microchannels

A man at age 28 was admitted to our hospital for recurrent spontaneous chest pain for 1 day in September 2016. He had a history of hypertension, dyslipidemia and surgical atrial septal defect closure. CAG showed less than 50% stenosis in proximal segment of LAD (Fig. 1A). The patient was treated with aspirin, nitrate, β-blocker and statin after discharge. He was readmitted to our hospital for paroxysmal chest pain for 1 month at age 35 in April 2023. The patient was diagnosed with unstable angina during multiple hospitalization as his vital signs were normal, and no obvious abnormality was found in electrocardiogram, chest X-ray, cardiac ultrasound and laboratory examinations including cardiac troponin I and creatine kinase MB isoenzyme.

CAG revealed severe stenosis in the proximal and middle LAD (90% stenosis near the septal branch) and twisting channels was seen in the diseased segments (Fig. 1B). With the aid of a microcatheter, a Fielder XT-A guidewire was crossed the lesion and exchanged to a workhouse guidewire. After low pressure dilation with a 2.0 mm balloon (6 atm) and OCT examination, a 3.0 × 36 mm stent was implanted in proximal and middle segment of LAD (Fig. 1C). OCT imaging were performed before stent implantation and demonstrated multiple lumens separated by fibrous tissue and the lumens shared the media and adventitia (Fig. 1D and E). There were two separated main channels in the diseased segments. The guidewire and the OCT catheter were located in the larger channel and no communication were found with the smaller one. The later originated from the proximal segment (Fig. 1D ①, ②), soon emitted multi-microchannels (Fig. 1D ③ to ⑦) and then converged (Fig. 1D ⑧). Again, it emitted multi-microchannels (Fig. 1D ⑨ to ⑭) and then converged (Fig. 1D ⑮ to ⑱). Both CAG and OCT indicated features of a WCA in the proximal and middle LAD. As CAG showed only mild stenosis in proximal LAD 7 years ago, we consider that the woven change of LAD was acquired and caused by the recanalization of thrombus. During one year follow-up, the patient had no adverse cardiovascular events or discomforts. This study was performed in accordance with the Declaration of Helsinki and was approved by the Ethics Committee of Fuwai Hospital. The patient provided written informed consent.

## Discussion

Here we show a natural history of WCA in LAD of a young man by serial CAG in a 7-year interval, and the lesion features were visualized by OCT imaging. Our case indicates an acquired cause of WCA. This supports the finding in another report, in which WCA development was observed in the right coronary artery by serial CAG in a 6-year interval, and the lesion characteristics were observed by intravascular ultrasound (IVUS) [4]. Till now, only these two case reports described the natural history of WCA by serial CAG.

Multiple theories have been proposed to explain the etiology of WCA, although the exact cause remains unknown [5]. The previous literature highlights two main theories regarding its pathology. One theory suggests that WCA is congenital and sporadic, with no reported cases indicating a genetic predisposition or hereditary cause [6]. Another theory proposes that WCA is an acquired condition, results from vasculitis disorders, spontaneous coronary artery dissection, recanalized thrombus, or Kawasaki disease [4, 7, 8], and it may also be linked to chronic conditions like rheumatic valvular heart disease [9]. It’s important to note that WCA can mimic these pathological states, so they should be included in the differential diagnoses when considering a potential case of woven coronary artery anomaly. Despite the unknown pathophysiology, some have postulated that certain growth factors involved in arteriogenesis and angiogenesis play a key role in the disorder’s underlying mechanisms [5].

Overall, the most commonly supported theory in the literature is that WCA is of congenital origin [5]. However, our case report challenges this theory. The OCT image features of WCA is summarized as follows: (1) Intertwined thin channels separated by fibrous tissue without cross-communications among them; (2) Undisrupted arterial wall integrity without dissection; and (3) High signal intensity and low signal attenuation [1]. Although it is not conclusive, no cross-communications is proposed as a key feature in distinguishing congenital WCA from other etiologies, such as thrombus recanalization [1]. In the current case of WCA which is acquired, we also found no cross-communications among the lager and the smaller channels by OCT. However, the smaller channel itself has multiple microchannels and cross-communications exist among them. Taking into account the limited number of published case reports, we consider that no cross-communications among the channels is just one type of WCA, thus could not be used as a criterion for differential diagnosis.

Among the acquired factors, we consider that the woven change in the LAD most likely resulted from recanalized thrombus based on the patient’s history, CAG and OCT image. Firstly, although not conclusive, our findings do not support healed SCAD as the etiology. SCAD is known as a nonatherosclerotic disease and women comprise 87–95% of the patients [10]. However, our patient is a 35-year-old male and the OCT image showed an intact media without the presence of false and true lumens. Furthermore, CAG indicated an atherosclerotic lesion in the same location 7 years ago. Secondly, Kawasaki disease (KD) could be ruled out as the patient had no past medical history related to KD and CAG did not show aneurysmal coronary abnormalities. Thirdly, arteritis was excluded. The patient underwent coronary computed tomography angiography (CTA) and thoracic CTA in local hospital in 2016, and no vasculitic changes were found. Finaly, CTO lesion with bridging collateral vessels may be ruled out. It is not rare that neovascular channels form in the CTO segments, but histologic studies show their diameters range from 160 to 230 μm [11]. The diameters of two main channels (about 2000 μm and 1000 μm, respectively) in the current case were much larger than that of neovascular channels in CTO lesions, and the antegrade blood flow in the LAD was Thrombolysis in Myocardial Infarction (TIMI) grade 3. Thus, the lesion is unlikely resulted from recanalization of a CTO. Consequently, recanalization of thrombosis is most likely to be the underline mechanism for this case. Taken together with the previously reported case [4], which also observed a natural history of WCA, we speculate that WCA is most likely an acquired coronary artery disease.

In the past, WCA was deemed to be a benign abnormality and usually do not need intervention [12]. It is now recognized that WCA may lead to ischemia and even myocardial infarction and surgical or percutaneous revascularization is indicated in such patients [1]. The literature review of case reports (37 cases) showed that 24 (50.4%) cases were single vessel disease, and 18 (48.6%) cases underwent revascularization. Among them, 5 (13.5%) cases received coronary artery bypass graft (CABG) and 13 (35.1%) cases underwent percutaneous coronary intervention (PCI) [1]. Thus, PCI is a therapeutic option for most of WCA patients with documented ischemia when the anatomy is suitable. We suggest two technical points when PCI is planned for WCA: (1) Use polymer sleeve CTO guidewires to cross the lesion with sliding and rotating technique. If it does not work, penetration technique with coiled CTO guidewires may be considered; and (2) Confirm that the multi-channels share the media and the guidewire locates inside the media by intravascular imaging, so that the stent could be safely expended without risk of perforation.

## Conclusion

This case report demonstrates that WCA may present with an acquired etiology, although congenital causes cannot be excluded for other cases.

### Electronic supplementary material

Below is the link to the electronic supplementary material.

Supplementary Material 1

## Data Availability

The datasets used and/or analyzed during this study are available from the corresponding author on reasonable request.

## Abbreviations

WCA: Woven Coronary Artery
CAG: Coronary Angiography
LAD: Left Descending Artery
